# Hepatitis B virus X protein inhibits apoptosis by modulating endoplasmic reticulum stress response

**DOI:** 10.18632/oncotarget.21630

**Published:** 2017-10-06

**Authors:** Jia Li, Jiang He, Yongming Fu, Xingwang Hu, Lun-Quan Sun, Yan Huang, Xuegong Fan

**Affiliations:** ^1^ Department of Infectious Diseases, Hunan Key Laboratory of Viral Hepatitis, Xiangya Hospital, Central South University, Changsha 410008, China; ^2^ Center for Molecular Medicine, Center for Molecular Medicine, Xiangya Hospital, Collaborative Innovation Center for Cancer Medicine, Central South University, Changsha 410078, China; ^3^ Key Laboratory of Molecular Radiation Oncology, Changsha 410008, China; ^4^ Department of Emergency, Xiangya Hospital, Central South University, Changsha 410008, China

**Keywords:** HBV, HBx, hepatocellular carcinoma, ER stress, apoptosis

## Abstract

Chronic Hepatitis B virus (HBV) infection is a major risk of hepatocellular carcinoma (HCC) worldwide. Hepatitis B virus X protein (HBx) is encoded by one of the four open reading frames of HBV, and is well known as an important coactivator for HBV replication and HBV-associated hepatocellular carcinogenesis. However, its role in keeping cells from apoptosis to promote HCC proliferation remains controversial. Here, we used HBx expressing HCC cells as a model, to investigate the mechanism of HBx-mediated cellular response to endoplasmic reticulum (ER) stress. We found that HBx protein was localized in ER lumen and interacted with GRP78 directly. This interaction resulted in suppression of eIF2α phosphorylation, inhibited expression of ATF4/CHOP/Bcl-2, and reduced cleavage of poly ADP-ribose polymerase (PARP) and level of γH2AX, thus preventing HCC cells from cell death and negatively regulating DNA repair. This study reveals a novel mechanism of the HBx-mediated oncogenesis and provides a basis for potential HBx-targeted therapeutic intervention of HCC.

## INTRODUCTION

Chronic Hepatitis B virus (HBV) infection is closely associated with the development of liver cirrhosis and then progression to hepatocellular carcinoma (HCC) [1]. Although HBV association with HCC has been well documented, the sophisticated mechanisms of HBV-mediated oncogenesis remain to be fully elucidated. Among the proteins translated from the open reading frames of HBV genome, HBx protein has been shown to play prominent roles in HBV-mediated carcinogenesis. HBx is mainly localized in the nucleus and mitochondria, and is a multifunctional transactivator that regulates host cell proliferation, metabolism, autophagy and senescence [2–5]. However, how HBx causes hepatocytes transformed or maintains malignancy of HCC remains still elusive.

Liver is the largest exocrine gland and detoxification organ in the human body, and the endoplasmic reticulum (ER) in hepatocytes has a complex functionality. Abnormal accumulation of unfolded proteins that exceed the capacity of the protein folding machinery will lead to a state of “ER stress”, which at the early phase stimulates unfolded protein response (UPR) to protect hepatocytes by inhibiting protein synthesis [6]. However, sustained or excessive ER stress induces apoptosis. To counter the HBV-induced ER stress, the virus must have an adaptive mechanism to maintain survival of the infected cells.

During ER stress, there are three major UPR stress sensors including PERK (PKR-like ER kinase), IRE1α (inositol-requiring enzyme 1α) and transcription factor ATF6. Under UPR, the diverse substrate repertoire of GRP78 enables it to function as a master regulator of the UPR by binding to and inactivating the three ER stress sensors, PERK, IRE1, and ATF6 [7]. During ER stress, increased levels of unfolded protein substrates lead to the sequestration of GRP78, freeing the sensors to initiate UPR signaling. ER stress leads to phosphorylation of PERK and its downstream signal molecule eukaryotic translation initiation factor-α (eIF2α), which then causes global translation attenuation, while paradoxically increases ATF4 and C/EBP-homologous protein (CHOP) expression. During prolonged ER stress, CHOP acts as a death-related transcription factor to promote transcription of the apoptosis associated genes such as Bcl-2, DR-5 and Puma, which lead to cell apoptosis [8, 9]. In another branch of the UPR signal pathway, activated IRE1α becomes oligomerized and autophosphorylated, leading to the activation of its cytosolic RNase domain and cleaves the mRNA encoding the transcription factor X box-binding protein (XBP1). Spliced XBP1 (XBP1s) translocates to the nucleus to induce the expression of its target genes, which participate in ER-associated degradation (ERAD) in order to alleviate ER load. Activated IRE1α also mediates the rapid degradation of a specific subset of mRNAs encoding plasma membrane and other secreted proteins in a XBP1 independent way to relieve acute ER stress [10]. When ER stress passes a critical threshold, IRE1α expands its RNase substrate repertoire to many other ER-localized mRNAs, driving cells into apoptosis [11]. The third classical pathway of UPR is mediated by ATF6. Under ER stress conditions, ATF6 is translocated into Golgi apparatus, where it is processed by site-1 and site-2 proteases to release a cytosolic fragment (ATF6f) and directly controls the upregulation of genes encoding ERAD components and XBP1 [12].

Many virus infections like Hepatitis C Virus, Herpes Simplex Virus, African Swine Fever Virus and cytomegalovirus, are already known to impose ER stress on the host cells [13–16]. They induce the UPR due to their demand on viral protein production, replication and cell survival. As to HBV infection, accumulated pre-S proteins and surface antigens in ER lumen have been identified as the cause of hepatocyte UPR [17]. Cellular localization of the viral proteins has been suggested to be one of the determinants for their functions. It has been reported that the large HBV surface antigens (HBsAg) with deletions at the pre-S1 and pre-S2 regions could be retained in endoplasmic reticulum and resulted in a strong ER-stress response to induce oxidative DNA damage and genomic instability in hepatocytes [18]. HBsAg of the HBV genotype G with filamentous morphology could also accumulate in endoplasmic reticulum and diminished activation of Nrf2, thus resulting in defects of liver regeneration [19]. Cho et al reported that HBx could induce the proliferation of hepatocellular carcinoma cells via AP1 over-expression as a result of ER stress [20]. However, the detailed mechanism whereby the HBV infected cells survive from the ER stress caused by HBV infection is still not fully understood.

In this study, we sought to investigate the impact of HBx on ER stress. We assessed the HBx cellular localization, effect of HBx on three UPR sensors, and explored potential mechanism whereby HBx relieves ER stress to promote HCC survival.

## RESULTS

### HBx is localized in ER

**Figure 1:**
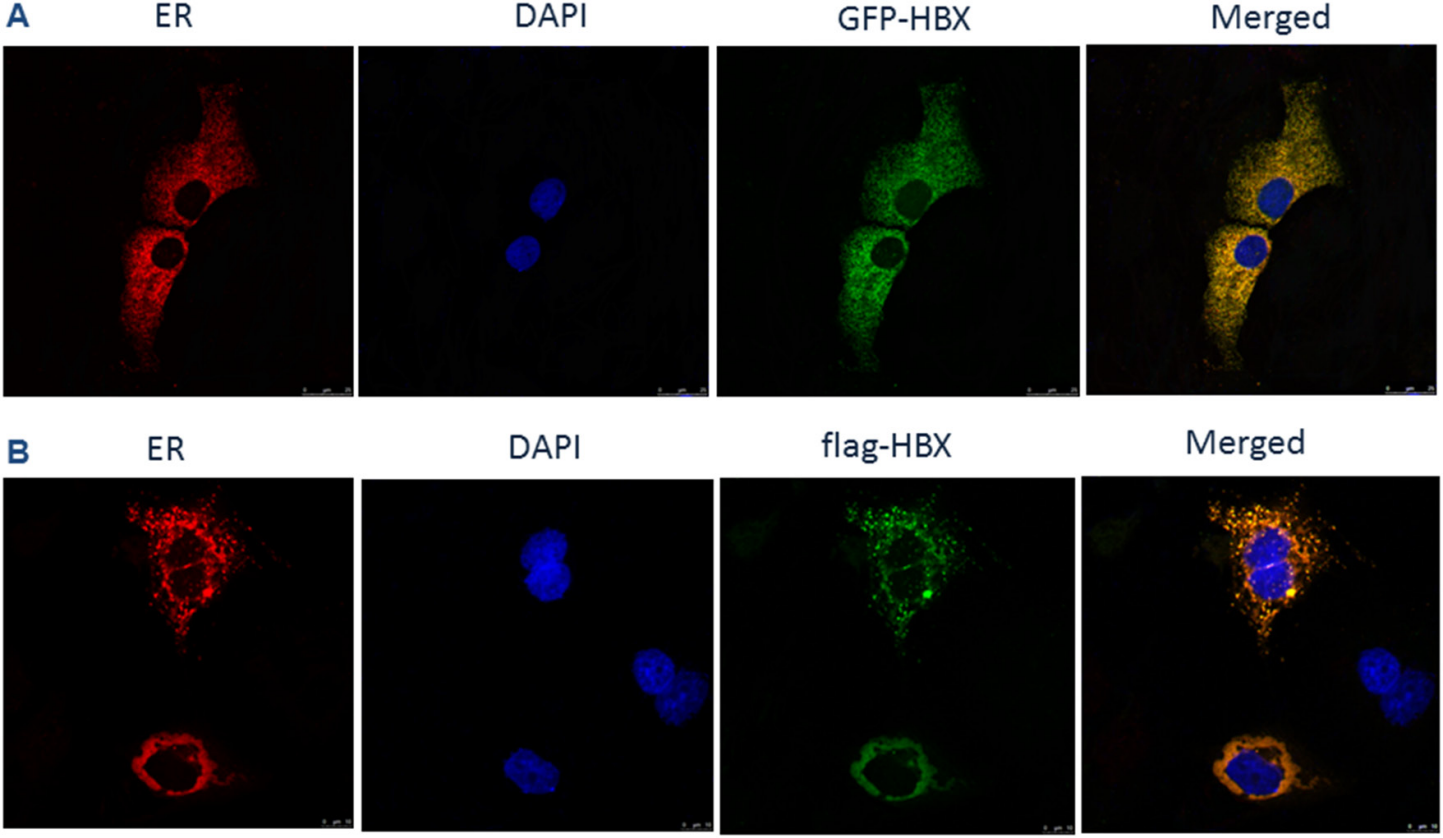
HBx localization on ER **(A)** HepG2 cells were co-transfected with pcDNA 3.1-GFP-HBX and pDsRed2-ER plasmids for 24h. Cells were rinsed and fixed. The coverslips were mounted and imaged using a laser scanning confocal microscope. **(B)** HepG2 cells were co-transfected with pcDNA 3.1-flag-HBX and pDsRed2-ER plasmids for 24 h. Cells were rinsed, fixed, permeabilized and blocked with BlockAid™ blocking solution. After labeling with anti-flag antibody overnight at 4°C, cells were washed in PBS and incubated with Alexa Fluor-conjugated secondary antibodies for 45 min at room temperature. The coverslips were mounted with DABCO anti-fade agent on glass slides and imaged using a laser scanning confocal microscope.

In HCC, the cancer cells need to overcome the chronic ER stress to sustain the pro-survival signals, in which HBx, as one of the key oncogenic proteins, may play a role. To test this hypothesis, we first examined whether HBx could be localized in endoplasmic reticulum. We co-transfected an HBx-GFP plasmid which expressed a green fluorescence HBx-GFP fusion protein, and an ER localization signal vector expressing red fluorescence (pDsRed2-ER Vector), to see if these two colors could be merged together. As shown in Figure 1A, the green fluorescence co-located with the red fluorescence. To further confirm this observation, we utilized a flag-HBx vector to co-transfect HepG2 cells with pDsRed2-ER vector and showed that, the flag and DsRed signals were co-localized in ER (Figure 1B). These data suggested that HBx protein was localized in the ER of HCC cells.

### HBx relieves ER stress by binding to GRP78

**Figure 2:**
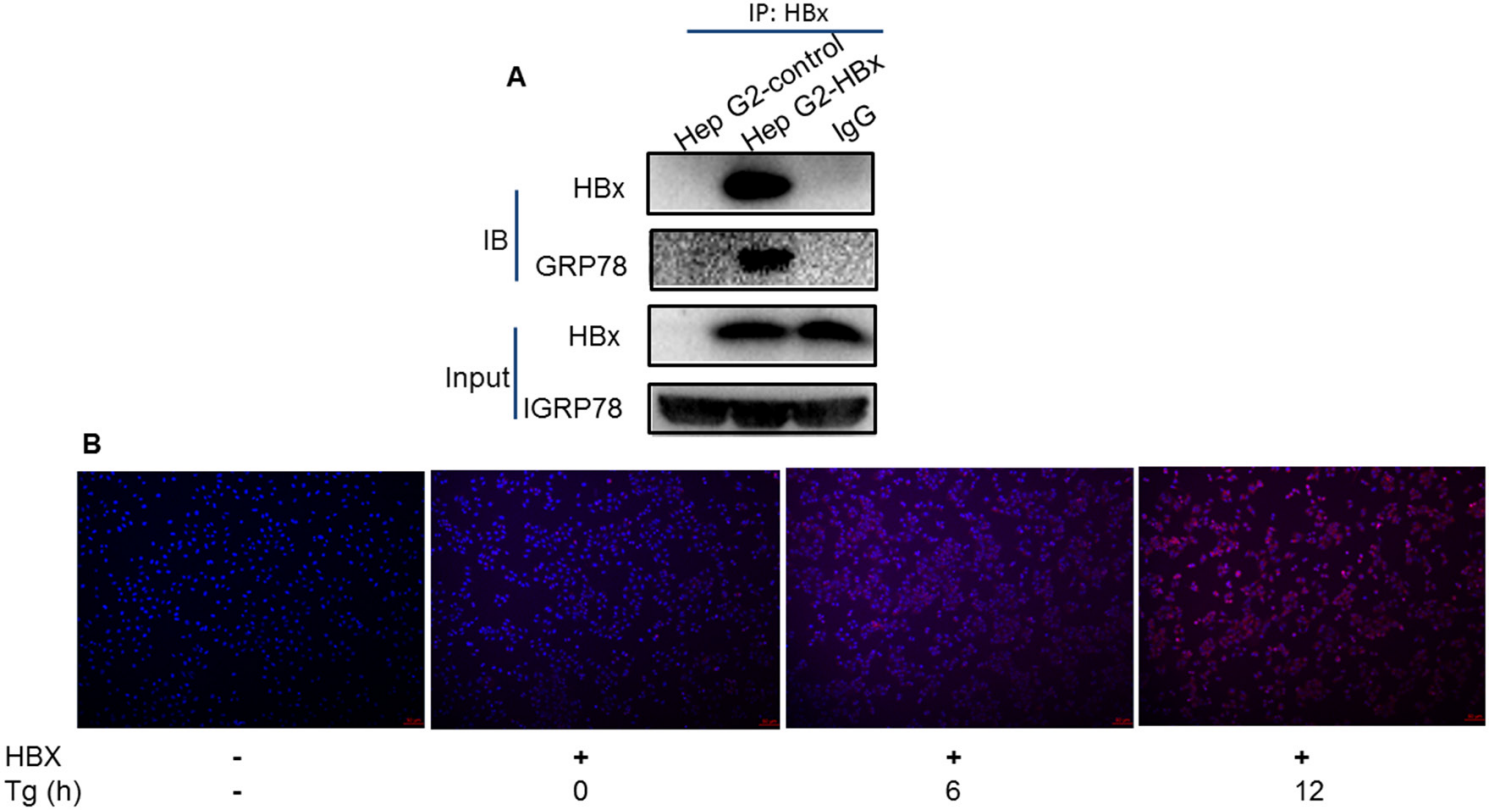
Direct interaction between HBx and Grp78 **(A)** Protein was extracted from the HepG2 cells expressing HBx or control cells and immune-precipitated with Anti-HBx antibody. The precipitates were subjected to immune-blotting by Anti-HBx and Anti-Grp78 antibodies. Loading control was indicated as Input. **(B)** Proximity ligation assay in HepG2 cells with or without HBx expression under Tg treatment up to 12 h. The red spots show sites of proximity ligation assay amplification reflecting the interaction between HBx and Grp78.

To examine the significance of HBx localization in ER, we next investigated if the HBx localized in ER could directly interact with GRP78 by co-immunoprecipitation assays in HepG2 stably expressing HBx protein. As shown in Figure 2A, HBx could be co-precipitated with GRP78. To further verify the interaction between HBx and Grp78, a Duolink proximity ligation assay was performed to help us visualize HBx and Grp78 interaction in cells. The results showed that the interaction between HBx and Grp78 indeed occurred in a time-dependent manner (Figure 2B).

### HBx promotes IRE1α and ATF6 pathways under ER stress

**Figure 3:**
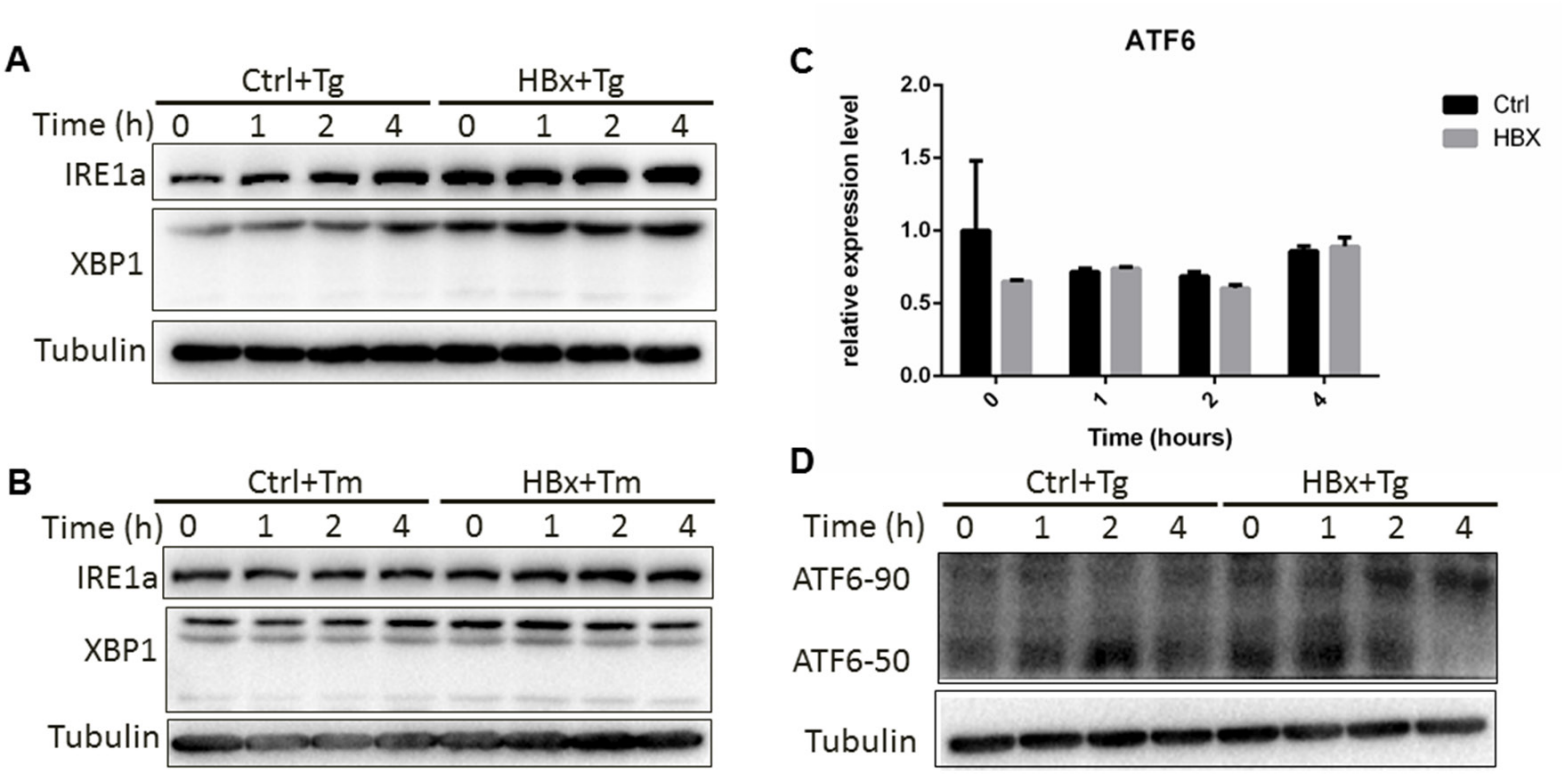
HBx impact on IRE1a and ATF6 pathways HepG2 cells expressing HBx or control cells were treated for the indicated time with Tg **(A)** or Tm **(B)**. The protein was extracted and subjected to Western blotting **(D)** and RNA was extracted for RT-PCR of ATF6 expression **(C)**.

Having demonstrated a direct interaction between HBx and GRP78, we next examined the effect of this interaction on ER stress responses. IRE1α pathway has been implicated in generation of pro-survival signals while the ATF6 are involved in production of the proteins needed for protein folding, transport and degradation [21]. We utilized a HepG2 cell stably expressing HBx and tested the ER signaling simulated with two ER stress inducers (Tg and tunicamycin Tm). As shown in Figure 3A and 3B, IRE1α was significantly increased in the presence of HBx, which companied with an increase of the XBP1s. For ATF6, we found that although HBx had no impact on the ATF6 mRNA level, expression of HBx actually could promote ATF6 activation at the late time point (Figure 3C and 3D). This data suggested that HBx might impact on IRE1α and ATF6 branches of ER response to promote cell survival.

### HBx modulates ER stress response by inhibiting eIF2α/ATF4 pathway

**Figure 4:**
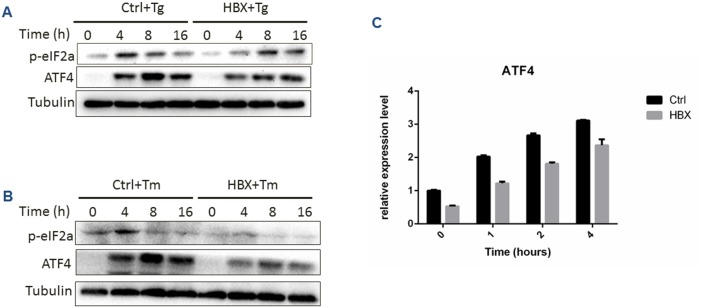
Effect of HBx expression on PERK branch under ER stress condition **(A)** HepG2 cells expressing HBx or control cells were treated for the indicated time with Tg (A) or Tm **(B)**, and p-eIF2a and ATF4 protein levels were measured by Western bots. Relative expression of ATF4 mRNA was detected by RT-PCR assay **(C)**.

In addition to IRE1α and ATF6 pathways, we also examined the effect of HBx on PERK signals, which is one of the important branches of UPR. Upon ER stress, PERK dimerizes, autophosphorylating and facilitates the phosphorylation of eIF2α. A phosphorylated eIF2α proceeds to stop further protein translation, as well as activate the transcription factor ATF4. Under Tg or Tm treatment, the control cells responded well to the ER stress while the HBx-expressing cells showed a significant delay and reduction of eIF2α and ATF4 expression (Figure 4A-4C), indicating that HBx could inhibit eIF2α/ATF4 pathway.

### Inhibition of apoptosis by HBx under ER stress

**Figure 5:**
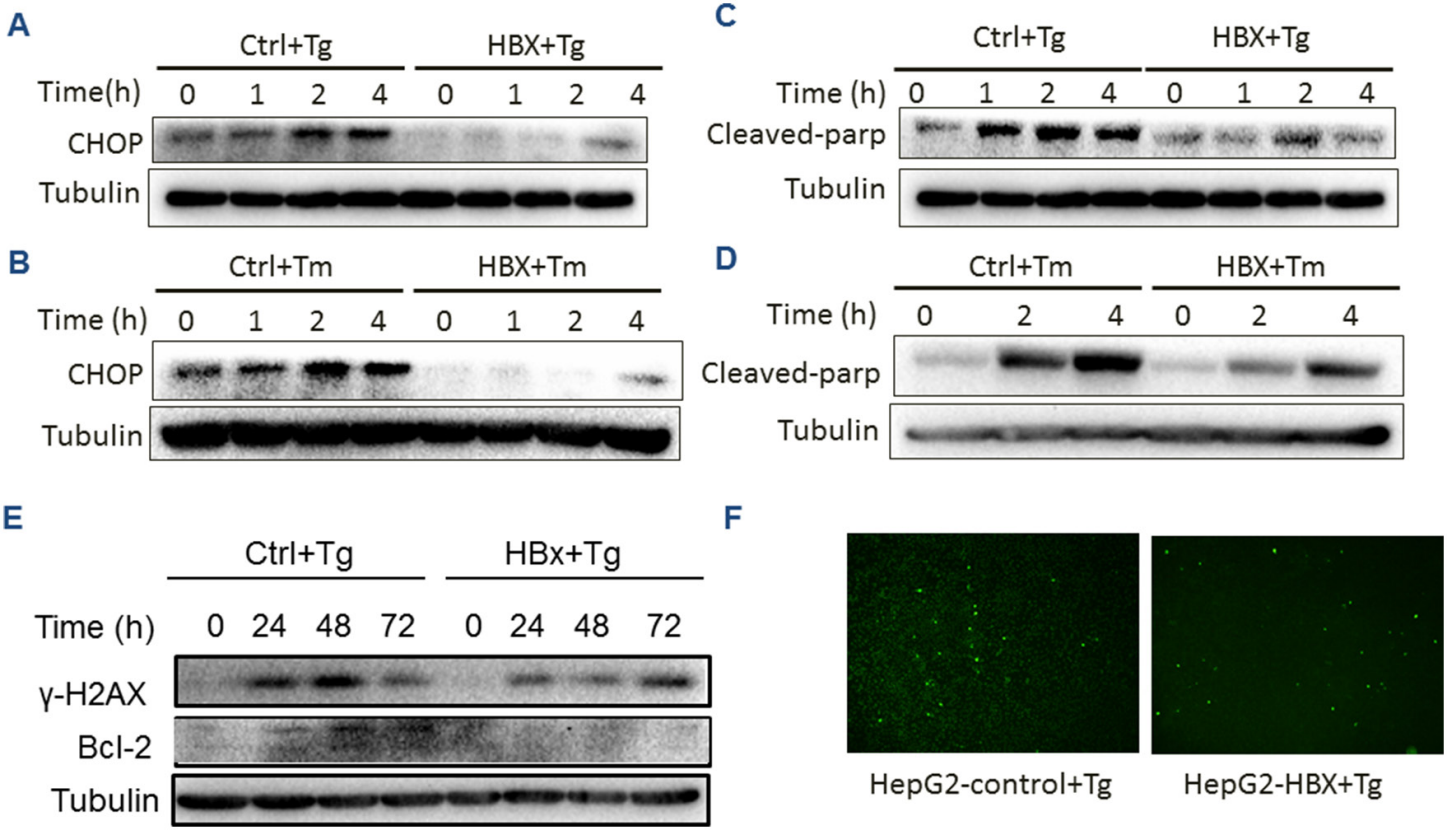
HBx inhibition of apoptosis and DNA repair HepG2 cells expressing HBx or control cells were treated for the indicated time with Tg **(A, C, E, F)** or Tm **(B, D)**. Expression of CHOP **(A and B)**, cleaved PARP **(C and D)** and γ-H2AX and Bcl-2 were measure by Western blots. The Tg-treated HepG2 cells were assayed for apoptosis by TUNEL staining (F).

Following prolonged stress, if not relieved, cell death can be initiated by the UPR. This process is predominantly mediated by ATF4 in the PERK down-stream molecules, which enters the nucleus, binds DNA and promotes the gene expression of CHOP to trigger apoptosis [22]. To verify the effect of HBx on the PERK pathway, we first examined the level of CHOP expression under ER stress conditions in HepG2 cells. The results showed that the CHOP level was significantly reduced in the presence of HBx, compared with the control (Figure 5A and 5B). Then the level of Bcl-2, one of the CHOP regulated anti-apoptotic genes, was found to be decreased in HBx-expressing cells (Figure 5E). At cellular level, the HBx-expressing HepG2 cells showed much less proportion of apoptotic cells under Tg treatment as measured by a TUNEL assay (Figure 5F). Interestingly, when the level of poly (ADP ribose) polymerase 1 (PARP-1), a key executer of DNA repair and apoptosis, was further assayed, we found that the expression of HBx markedly inhibited the level of the cleaved PARP-1 (Figure 5C and 5D), which suggested that HBx inhibited the PERK pathway, avoiding the activation of ATF4-mediated DNA repair. This was further confirmed by measuring level of γ-H2AX (Figure 5E). To exclude the possibility that the observation was cell-specific, we used another HCC cell line Huh7 to verify the results, showing that HBx-expressing Huh7 cells exhibited similar molecular and cellular changes to HepG2 under Tg treatment (Supplementary Figure 1). Together, these data provided evidence of HBx effect on DNA repair and apoptosis.

## DISCUSSION

Human oncoviruses appear to be necessary but not sufficient to cause cancer and are rarely fully oncogenic per se. This indicates that within the context of multistep carcinogenesis, viral infection provides only a subset of the required oncogenic hits [23]. In HCC, HBV establishes chronic infection, and when accompanied by hepatitis, hepatocellular destruction triggers regeneration and fibrosis, which can evolve into cirrhosis and HCC. During the chronic phase of HBV infection, HBx directly promotes HCC by altering host gene expression, while immune-mediated inflammation contributes indirectly to tumorigenesis [24]. How HBx alters the host gene expression has been the focus of many intensive investigations. In the present study, we found that HBx could directly interact with Grp78, perturb ER stress response and re-set the host gene expression, which resulted in the acquisition of anti-apoptotic capacity by hepatocytes. This supports the notion that HBV evades growth suppression and avoids immune destruction by blocking apoptosis, providing a survival and growth advantage for HBx expressing hepatocytes [25].

IRE1α is a dual function protein with both kinase and endoribonuclease properties. While the kinase activity is responsible for activating ASK1 and JNK to promote cell death [26], the endoribonuclease activity promotes the splicing of a critical pro-survival gene XBP1 [27]. In this study, we observed that HBx could suppress PERK branch, but activate IRE1a branch although HBx was shown to directly bind to Grp78. We speculate that under the stress condition, HBx binds GRP78, leading to inhibition of PERK branch to avoid apoptosis on the one hand, and activation of IRE1α branch to further provide the survival advantage on the other hand.

Virus replication presents the host cell with large amounts of exogenous genetic material and unusual structures. The battle between virus and host generates a “genomic conflict”. The host attempts to limit viral infection and protect its genome, while the virus deploys tactics to eliminate, evade or exploit aspects of cellular defense, among which the host ER stress response-triggered DNA repair mechanism appears well-suited machinery used by virus [28, 29]. In this study, we found that HBx could inhibit PARP-1 that regulates both DNA repair and apoptosis, suggesting that HBx may function as a mediator to restrict host DNA damage response, thus maintaining survival of the HBV-infected cells.

In conclusion, our study suggests a novel mechanism by which HBV-infected cells escape the ER stress-induced apoptosis via attenuation of PERK activation and restriction of DNA repair response. Thus, direct targeting of HBx in combination with the standard therapy may present a potential strategy for clinical management of HCC.

## MATERIALS AND METHODS

### Cell lines, plasmids, transfection and generation of stable cell lines

HepG2, Huh7 and HEK293T cells were from American Type Culture Collection (ATCC) and maintained at 37°C under 5% CO2 in Dulbecco's modified Eagle's medium (DMEM) added with 10% fetal bovine serum (FBS), 100U/ml penicillin and streptomycin.

PcDNA 3.1-Flag-HBX, pcDNA 3.1-Flag and pDsRed2-ER plasmids were purchased from Addgene (Cambridge, MA). Transient transfection was performed using X-tremeGENE HP DNA Transfection Reagent (Roche, Shanghai).

Lentiviral transduction system (TaKaRa, Beijing) was used for stable cell line construction. The intact HBX gene was amplified from HepG2 2.15 cells and then inserted into the pLV –cDNA vector. The pLV-cDNA containing HBX gene was co-transfected with three helper plasmids (Gag-Pol, Rev and VSV-G) for lenti-cDNA viral stocks generation. After 72 hours transduction of lenti-cDNA into HepG2 and Huh7 cells respectively, we use BSD for stable cell line selection.

### Reagents and antibodies

Chemical reagents and kits used in this study included Thapsigargin (SIGMA-ALDRICH), Tunicamycin (Beyotime), ApopTag^®^ Fluorescein *In Situ* Apoptosis Detection Kit (Merck Millipore), and PrimerSTAR^®^ GXL DNA Polymerase (TaKaRa).

Primary antibodies used in this study included the following: Phospho-eIF2α (Ser51) (CST), ATF4 (CST), CHOP (CST), p58IPK (CST), Cleaved PARP (Asp214) (CST), HSPA5 (Proteintech), HBx (XIAMEN INNOVAX BIOTECH), IRE1a (CST), XBP1 (Abcam). The secondary antibodies used for Western blot were Anti-rabbit IgG, HRP-linked (CTS), Anti-mouse IgG and HRP-linked (CST).

### Western blot analysis

Cells were washed three times with ice-cold PBS and were lysised in RIPA buffer with 10% cocktail on the ice for 30 min. Lysates were centrifuged at 14000 rpm for 20 min at 4°C. Equal amounts of proteins were run on 10-12% SDS-PAGE gel electrophoresis and then transferred on to PVDF membranes (Millipore). Primary antibodies were incubated in 4°C overnight and second antibodies were incubated at room temperature for 1 hour. ECL Western blotting kit was used for protein detecting according to manufacturer's instruction.

### Real-time qRT-PCR

To analyze the expression levels of ATF4 and ATF6 genes, total cellular RNA and subsequent complementary DNAs were prepared. The RNA levels of the genes were quantified by real-time qRT-PCR using the primers were as follows:

ATF4 forward, 5’-TTCTCCAGCGACAAGGCT AAGG-3’;

ATF4 reverse, 5’-CTCCAACATCCAATCTGTCC CG-3’;

ATF6 forward, 5’- CAGACAGTACCAACGCTT ATGCC-3’;

ATF6 reverse, 5’-GCAGAACTCCAGGTGCTTG AAG-3’.

Real-time qPCR was conducted by using an ABI PRISM 7100 Sequence Detection System (Applied Biosystems).

### Immuno-precipitation

Cells were fixed with 4% PFA for 15 min and lysed in RIPA buffer. Protein A/G magnetic beads were first suspended in binding buffer (50 mM Tris, 150 mM NaCl, 0.1%-0.5% Triton 100 or Tween 20, pH 7.5) and then incubated with 5 μg Grp78 or Hbx antibodies for 1 h at room temperature with end-over-end rotation. After the supernatant was removed, 200 mg of cell lysate was added to each tube, which was incubated with rotation for 10 min at room temperature. The immune-precipitated proteins were released by boiling for 5 min at 95°C in dodecyl sulfate sodium salt -Polyacrylamide gel electrophoresis (SDS–PAGE) sample buffer. The magnetic beads were removed with a magnetic separator before the samples were loaded onto a 12% SDS–PAGE gel.

### Immunofluorescence

Cells were quickly rinsed with pre-warmed PBS and fixed with 4% paraformaldehyde (PFA) in PBS for 20 min at 37°C, permeabilized in 0.5% Triton X-100 for 20 min and blocked with BlockAid™ blocking solution (Thermo Fisher) for 1 h. After labeling with primary antibodies overnight at 4°C, cells were washed in PBS and incubated with Alexa Fluor-conjugated secondary antibodies for 45 min at room temperature. All antibody incubations were performed in BlockAid™ blocking solution. The coverslips were mounted with DABCO anti-fade agent on glass slides and imaged using a laser scanning confocal microscope (TCS Sp8 X&MP; Leica) equipped with a 63x/1.4 numerical aperture oil-immersion objective (Leica) objective.

### Duolink proximity ligation assay

The *in situ* proximity ligation assay was performed using a Duolink^®^ *In Situ* Red Starter Kit for Mouse/Rabbit (DUO92101, Sigma) according to the manufacturer's instructions. Briefly, cells were seeded onto coverslips and circled with a hydrophobic pen the day before the experiment. After treatment, the cells were fixed, permeabilized, blocked, and then incubated with primary antibodies at 4°C overnight. After washing, the oligonucleotide (Minus and Plus)-conjugated secondary antibodies were added and incubated for another hour at 37°C. Subsequently, cells were washed and incubated with ligation solution for 30 min at 37°C. The ligated nucleotide circles were amplified using polymerase via the addition of amplification solution and incubation for 100 min at 37°C. The slides were washed briefly, and Duolink^®^ *In Situ* Mounting Medium with DAPI (DUO82040, Sigma) was added to each sample to stain cell nuclei for fluorescence microscopy. The visualized fluorescence spots represented the clusters of protein-protein interactions.

## SUPPLEMENTARY MATERIALS FIGURES AND TABLES


