# Supplementary material for: Hepatitis B virus X protein inhibits apoptosis by modulating endoplasmic reticulum stress response
Source: Oncotarget. 2017 Oct 6;8(56):96027–34. doi: 10.18632/oncotarget.21630 (PMC5707078; doi:10.18632/oncotarget.21630)
Supplement: Supplementary file 1 [file oncotarget-08-96027-s001.pdf]

Hepatitis B virus X protein inhibits apoptosis by modulating endoplasmic reticulum stress response

SUPPLEMENTARY MATERIALS

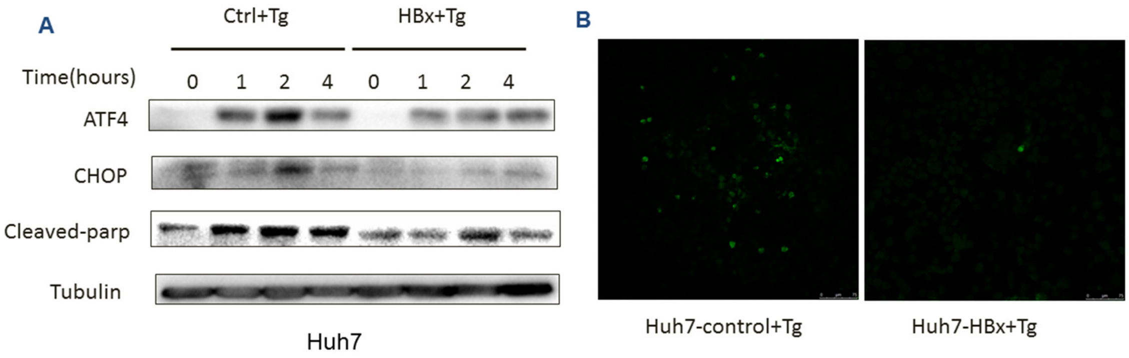

Supplementary Figure 1: Effect of HBx on ER stress responses in Huh7 cells. **(A)** Western bots for detection of expression of ATF4, CHOP and cleaved PARP genes in HBx-Huh7 and Vector-Huh7 cells treated with Tg. **(B)** TUNEL assay for HBx-Huh7 and Vector-Huh7 cells treated with Tg.
